# Supplementary material for: Are carbon emissions trading and green financial instruments synergistic? -Comprehensive quantitative research based on content analysis
Source: PLoS One. 2024 Mar 7;19(3):e0298601. doi: 10.1371/journal.pone.0298601 (PMC10919601; doi:10.1371/journal.pone.0298601)
Supplement: S1 Data — (ZIP) [file pone.0298601.s001.zip › Data/地级市Z_F工作报告.zip!/║■▒▒/╨ó╕╨╩╨/└┤╫╘╣1⁄2╓┌║┼í╛┬φ┐╦╩2╛▌═°í┐_2014.docx]

各位代表：

　　现在，我代表市人民政府向大会报告工作，请予审议，并请各位政协委员和列席人员提出意见。

　　一、2013年工作回顾

　　过去的一年，面对宏观经济下行和特大干旱等复杂严峻局面，市政府在省委、省政府和市委的正确领导下，紧紧依靠全市人民，以党的十八大和十八届三中全会精神为指导，按照“竞进提质、效速兼取”的总要求，围绕推进“五个跨越”、建设“五个城市”，改革创新、开拓进取，勇于担当、攻坚克难，全市经济保持稳中有进、稳中向好的发展态势，社会保持和谐稳定，主要经济指标在全省位居前列，增幅高于全省和武汉城市圈平均水平。预计实现生产总值1230亿元，增长10.5％；完成全社会固定资产投资1257亿元，增长28.2％；社会消费品零售总额598亿元，增长12.5％；地方公共财政预算收入89.1亿元，增长28.1％。

　　——产业实力明显提升。工业经济量增质好。预计规模以上工业实现增加值580亿元，增长13.6％。“三大工程”强力推进，净增规模以上工业企业102家，达到1052家；净增销售过亿元企业130家，其中过10亿元企业4家。新增高新技术企业32家，高新技术产业增加值增长18.5％。24个优势特色产业集聚区实现主营业务收入1527亿元，增长30.9％。与工信部合作开展40家小微企业“诊断”试点，小微企业管理水平、创新发展能力提升。新增市场主体7.1万户。建筑业增加值达到67亿元。农业经济保持稳定。夺取了有气象记录以来特大干旱斗争的全面胜利，粮食总产实现“十连增”。支持农业产业化龙头企业发展，新增农业产业化省级重点龙头企业19家，规模以上农产品加工业产值达到1026亿元。实施农田水利基本建设项目147个，完成投资20亿元。农业机械化水平提高到72.2％。灾害性天气监测预报预警系统建成投入使用。新解决25万人安全饮水。基本解决5.3万人脱贫。圆满完成第三轮“三万”活动。第三产业加快发展。以孝感为中心、县市为节点的区域物流网络建设加快，新增“限上”企业108家。新增贷款达到108亿元，孝感连续11次获评省金融信用市州。接待游客1400万人次、旅游综合收入84亿元，分别增长18.2％、19％。

　　——发展后劲明显增强。项目建设力度加大。强力推进事关孝感发展全局的100个重大工程项目建设，完成年度投资392.7亿元。在建亿元以上项目382个，其中10亿元以上项目30个。争取中央、省专项资金62亿元。一批交通、电力、通讯等基础设施项目建设加快。对外开放程度加深。新引进投资过亿元项目57个，其中10亿元以上项目16个。中国兵器装备集团、中国建筑工程总公司、中国交通建设集团、广东物资集团、马瑞利集团（意大利）等中外500强企业落户孝感投资。工信部五所华中分所落户孝感。外贸出口7.4亿美元，增长16％；实际利用外资2.75亿美元，增长15.8％。8家企业获准在境外投资，外派劳务人数居全省第一。“回归创业工程”领域拓宽，项目增多。市直“三区”发展速度加快。支持市直“三区”跨越式发展，下放市级行政审批权力113项。高新区探索建立快速高效落实机制，93个项目开工、28个项目投产。东城新区20平方公里基础设施建设启动，26个重点项目顺利推进。临空经济区纳入全省“大临空”规划，累计基础设施建设投入10亿元，启动建设物流项目4个。双峰山旅游度假区启动20里生态画廊规划建设，旅游公路建成通车。县域经济发展亮点纷呈。孝南规模以上工业企业突破100家，汉川财政总收入突破20亿元，应城新增市场主体达到1.3万户，云梦外贸出口实现翻番，安陆医药食品产业集群产值突破100亿元，大悟地区生产总值突破100亿元，孝昌产业链招商引进光电配套项目16个。

　　——城乡面貌明显改善。孝感市城市总体规划通过评审，“全域规划”体系不断完善。城镇化率提高到51％。孝感城区建设提速。实施城建项目146个，完成投资87.8亿元。人民广场二期、槐荫公园二期、八一水厂、污水处理厂二期等工程基本建成，107国道京广立交桥加固改造工程完工，长征北路、体育东路等市政维修工程进展顺利，槐荫公园三期、城际铁路站前广场开工建设。县市城区建设提质。6个县市城区共完成市政基础设施投资34.7亿元，新增建成区面积26.4平方公里。“美丽孝感”建设提升。“四城同创”长效机制初步建立，“四项行动”有序展开，“六大秩序”专项整治效果明显。文明卫生村镇、“一线一点”，建设步伐加快。编制完成王母湖、野猪湖、白水湖、汈汊湖等湖泊保护规划。全市城镇集中式饮用水源地水质达标率100％，森林覆盖率提高到20.6％。孝感城区新增绿地面积100万平方米，空气质量优良天数达到312天。实施减排项目65个，关闭高污染、高耗能企业17家，万元生产总值综合能耗、二氧化碳和主要污染物排放量完成省定目标任务。

　　——改革创新明显推进。坚持“三情”结合找科学有效路径，在市委统筹下，编制完成《大力推进“五个跨越”、加快建设“五个城市”纲要》。“两型”社会综合配套改革试验区9大领域38项改革试验走在武汉城市圈前列，大别山试验区基本实现“三年明显变化”目标。“十大融资办法”有效运用，15家企业启动“新三板”挂牌工作，7家企业在“四板”成功挂牌。孝感城区财税征管体制进一步完善。“营改增”试点全面启动。资源性产品价格改革稳步推进。“七大土地增容措施”用足用活，重大项目建设用地得到基本保障。推广土地适度规模经营，全市土地流转面积达到103万亩，占耕地面积的26.1％。社会管理方式不断创新，开工建设“三类”新社区82个，236个社区完成“1+X”综合服务中心规范化建设。城镇网格化管理和小区物业管理“两个全覆盖”领域拓宽。“天网工程”顺利实施。统治信访维稳“六大行动”深入开展，“十大工作机制”健全完善。机构改革有力推进，事业单位分类改革、食品药品、卫生计生、广播电视、新闻出版等机构改革全面启动。

　　——民生保障明显强化。教育、卫生、社保等民生支出占地方公共财政预算支出比例分别提高到21.4％、10.6％、14.9％。预计城镇居民人均可支配收入19810元，增长9.5％；农民人均纯收入8987元，增长12.5％。居民消费价格总水平保持基本稳定。政府承诺的“十件实事”顺利推进。社会保障取得新进展。实施“双助”行动，新增城镇就业5.4万人。城乡居民养老保险、医疗保险基本实现全覆盖。新改扩建农村福利院22所，建成示范性城乡居家养老服务中心62个。完成农村危房改造4436户。启动住房保障“三房并轨”试点，住房公积金扩覆1.7万人。社会事业取得新进步。孝感列入国家中小学教育质量综合评价改革首批试点城市，孝南、汉川、云梦分别通过教育部义务教育均衡督导评估，61所农村义务教育标准化学校建设顺利推进，8所市直学校改扩建工程开工建设。以城乡大病医疗保险为重点的医改工作深入推进。应城、孝昌县级公立医院综合改革进入全省第一方阵。出生人口性别比稳步下降。孝感荣膺“中国孝文化之乡”，成功举办“魅力孝感20年暨中华孝文化旅游节”，楚剧展演活动列为第二批全国公共文化服务示范创建项目。首部《孝感市志》出版发行。孝感成为国家知识产权试点示范城市，连续五年荣获“全国科技进步先进市”。精神文明建设取得新成效。“至孝至诚、图强图新”的孝感城市精神深入人心，涌现出“最美植保站长”周小贺、“最美社区警察”吴和平等一批先进典型。

　　——自身建设明显加强。全市政府系统深入开展“转作风、抓落实、促跨越”活动，求真务实、干事创业精神进一步增强。作风转变有力推进。严格执行中央、省委、市委作风建设规定，“三短一简一俭”有效落实，“四风”问题有效整治。推行机关“大科室”制改革，开展审批“三集中三到位”，减少内设机构226个。依法行政有力推进。主动接受人大、政协监督，82件人大代表建议、234件政协提案全部办结。开展相对集中行政复议权试点改革，加大“两法衔接”力度，顺利通过“六五”普法中期检查。廉政建设有力推进。突出重点对象、重点领域、重点环节，廉政风险防控机制初步建立。完成145项政府投资项目全程跟踪审计，审减资金2.5亿元。组织开展涉及群众切身利益的专项检查监督。

　　各位代表，过去一年成绩的取得，是省委、省政府正确领导、高度重视的结果，是市委统揽全局、科学决策的结果，是市人大、市政协支持配合、有效监督的结果，是全市广大干部群众同心同德、团结奋斗的结果。在此，我代表市人民政府，向所有参与、支持、关心孝感经济社会发展的各界人士，表示衷心的感谢并致以崇高的敬意！

　　在充分肯定成绩的同时，也要清醒地看到存在的问题和不足，主要是：经济总量、发展质量，与建设武汉城市圈副中心城市、打造“湖北的苏州”和“魅力孝感”战略目标还不相匹配；经济社会发展不均衡的问题仍然存在，民生事业欠账仍然较多；面临的改革任务更艰巨，矛盾和问题更复杂，成本和难度更大；干部作风有待改进，执行力、落实力还有差距。对于这些问题，我们将在今后的工作中切实加以解决。

　　二、2014年工作安排

　　2014年是贯彻落实党的十八届三中全会精神、全面深化改革的开局年，是实现全面建成小康社会阶段性目标的关键年。今年政府工作的总体要求是：坚持以邓小平理论、“三个代表”重要思想、科学发展观为指导，深入贯彻落实党的十八大、十八届三中全会和习近平总书记系列讲话精神，贯彻落实省委省政府和市委重大决策部署，坚持增量提质、改革创新，更好发挥政府作用，着力转变政府职能，着力激发市场社会活力，着力完善体制机制，着力保障改善民生，按照加快建设武汉城市圈副中心城市、打造“湖北的苏州”和建设“魅力孝感”目标，全面推进“五个跨越”、加快建设“五个城市”，开创孝感第三次创业新局面。

　　2014年主要预期目标是：生产总值增长11％左右；规模以上工业增加值增长14％左右；全社会固定资产投资增长25％左右；社会消费品零售总额增长14％以上；地方公共财政预算收入增长16％；城镇居民人均可支配收入、农民人均纯收入分别增长10.5％、12％；城镇新增就业4.3万人；人口自然增长率控制在6.5‰以内；居民消费价格涨幅控制在4％以内；单位生产总值综合能耗下降、二氧化碳和主要污染物排放量减少达到省定目标。

　　（一）致力于增强发展活力，加快推进改革创新

　　深化重点领域和关键环节改革。不断解放思想，勇于开拓创新，以改革促发展。紧紧围绕加快建设“五个城市”，正确、准确、有序、协调推进改革。正确处理政府与市场关系，强化市场主体地位，充分发挥市场在资源配置中的决定性作用。加快政府职能转变，推进事业单位分类改革，更好营造市场环境，激发经济发展内生动力。坚持城乡统筹发展，创新基础设施、公共服务两个“六张网”建设机制，健全农村土地流转市场，逐步破除城乡二元结构。构建现代公共文化服务体系，创新文化产业发展体制机制，加强孝文化研究和挖掘，不断扩大孝文化传播力和影响力。发挥武汉城市圈“两型”社会试验区、大别山试验区、国家高新区三个金字招牌的优势，加快推进一批综合改革试验。统筹推进“五个全覆盖”，提升社会治理能力，缩小城乡差距，建立公平、正义、法治社会。

　　推进创新创造创业。建设国际科技合作（孝感）创新园，建好“国家级军民结合产业示范基地”、“国家新型工业化产业示范基地”，培育高新企业孵化器、加速器，实施高新企业创业和培育工程，推进国家知识产权试点城市建设。深化产学研用合作，加快技术成果转化。鼓励企业进入资本市场融资。支持三江集团、华中光电加快民品发展。坚持政策引才、事业留才、环境聚才。制定实施“三支队伍”三年培养计划。坚持企业创新与全民创业并举，大力实施“回归创业工程”，创建“创业型城市”。

　　加强对外开放与合作。把招商引资作为扩大开放的主攻方向，用增量盘活存量，以外力激发内力。把握招商规律，提高招商能力，敢于利用优质资产、优势品牌开展合作，把资源向好项目倾斜。有效承接产业转移，加强产业链招商，实现集约集群发展。重奖引进重大项目的有功之臣。力争全年内联引资增长15％，引进过亿元项目65个,实际利用外资增长12％。继续实施外贸“三项工程”，壮大出口企业实力，外贸出口增长10％。深化区域合作交流，推进汉孝一体化建设，主动融入长江经济带、汉江经济带。加强友好城市经济文化交流。

　　（二）致力于提高经济发展质量，加快推进产业转型升级

　　推进新型工业化。实施“三大工程”，以工业的提质增效实现经济总量的提速扩容。深入推进“市场主体增量提质行动”，做多做强市场主体，净增规模以上工业企业80家、孵化企业1000家。构建骨干企业提升、中小企业成长、小微企业孵化梯次培育机制，做实品牌质量战略、中小企业公共服务、“智力支持+政银企集合贷”扶持三大平台。落实企业“直通车”服务制度，强化工业经济要素保障。加大园区基础设施建设力度，提升园区政务商务服务水平，促进各类生产要素向园区集中、向支柱产业聚集。鼓励龙头企业延伸产业链条，壮大产业集群，24个优势特色产业集聚区主营业务收入增长20％。大力培育节能环保、新能源、新材料等战略性新兴产业。深化“两化”融合试点，加快建设孝感软件产业园，全面建设“智慧孝感”。支持建筑企业对外发展，做好建筑劳务维权服务。

　　提升农业现代化。以“三农三化”促进现代农业加快发展。稳定粮食面积，优化粮食结构，确保粮食总产稳定在45亿斤左右。扩大农业板块基地规模，提升建设标准。抓好水利建设、农业综合开发、土地整治，发展设施农业、特色农业。促进农机与农技融合配套。继续推进“四个一批”工程，新增农产品加工产值过10亿元企业2家以上，农产品加工总产值增长15％以上。引导承包经营权向专业大户、家庭农场、农民专业合作社、农业企业流转，鼓励发展规模经营。扎实推进“一线一点”建设。实施精准扶贫，解决3万人基本脱贫。开展好新一轮“三万”活动。

　　壮大现代服务业。以“三个加快”推进服务业发展提速、比重提高、水平提升，把服务业打造成孝感跨越发展的新引擎。编制服务业发展规划，出台支持第三产业发展意见。加快发展生产性服务业。着力建设一批现代物流园区。支持民生银行、汉口银行在孝感设立分支机构，改制重组县市农商行，新设2-3家村镇银行，推进多层次资本市场建设。大力发展服务外包、信息咨询、科技研发、电子商务等新兴业态。加快发展生活性服务业。优化城乡商业网点布局，推进超市进社区，新增“限上”企业120家。积极发展家政等服务业，探索推进养老产业、健康服务产业。加快发展文化旅游业。编制完成全市文化旅游产业发展规划。挖掘孝文化特有内涵，开发孝感民俗文化资源，强化旅游项目支撑，推进文化与旅游深度融合，接待游客人数、旅游总收入分别增长17％、18％。

　　推动重大项目建设。按照提质、增效、升级要求，主动转变投资增长方式，促进产业转型升级。继续抓好事关孝感发展全局的100个重大项目建设，筑牢发展底盘。准确把握中央和省投资政策导向，积极谋划一批发展后劲强、带动作用大、综合效益好、打基础利长远的好项目。力争普惠性项目获得更多份额，个性化项目取得新突破，项目库滚动保持1.5万亿元投资规模。加快推进武汉城市圈环线高速、麻竹高速、107国道改造、孝感火力发电等重大基础项目建设。深化领导包保项目责任制，优化项目落户环境，新开工亿元以上项目186个，其中过10亿元项目20个。结合“十二五”规划中期评估，优化完善发展举措，谋划“十三五”发展规划。

　　（三）致力于拓展发展空间，切实加强区域协调统筹

　　推进新型城镇化健康发展。坚持以人为本，推进以人为核心的城镇化。城镇化率提高到52.1％。提升规划水平。优化城镇化布局和形态，丰富完善“1531”城镇空间体系，深化细化孝感中心城区总体规划，有序推进重点镇、示范村（社区）和重点项目规划。提升建设水平。推进孝感城区“六大组团”建设，逐步提升城市功能，有序拓展城市空间。按照延续城市历史文脉、保持孝文化特色要求，稳妥推进旧城区改造。围绕国家园林城市创建攻坚年，重点推进槐荫公园三期、澴川公园、邓家河中央湿地公园、仙女湖公园等绿地建设，新增绿地面积150万平方米。加快6个县市城区扩容提质，扎实开展“四化同步”乡镇试点，积极推进30个重点镇、100个中心村建设。落实放开城市落户限制的具体措施，促进人口合理有序流动和农村转移人口市民化。提升管理水平。按照创建时间节点，完成“四城同创”年度目标。加快推进城市管理体制机制改革，实行城市综合管理第三方考核评价问责，实施城市交通畅通工程。

　　推进市直“三区”加快发展。创新管理体制机制，推进“扁平化、模块化、流程化、绩效化”管理。推动高新区做强做大高端装备制造、光电子信息、食品加工等产业集聚区。强化科技创新的政策、金融、人才、平台支撑，提升产业核心竞争力，净增规模以上工业企业20家、高新技术企业10家以上、在孵科技创业企业30家。推动临空经济区抢抓“大临空”建设机遇，创新对外开放合作机制，加快推进基础设施、现代产业、城市社区三大建设，实现三年“出形象”的工作目标。推动双峰山生态文化旅游度假区建设，整治景区周边、沿线采石场，提升景区整体形象，夯实创建5A景区基础。

　　推进县域经济竞相发展。把加快发展县域经济作为提升孝感区域竞争力的重要举措，突出县域产业重点，强化县域发展支撑。支持孝南加快纸业扩能，建成百亿产业集群；汉川加快优势产业集聚，五大优势产业增加值达到160亿元；应城深度挖掘盐化、石膏资源，构建盐化为基、多元发展产业格局；云梦加快“一区三园”建设，建设云孝一体化先行区；安陆加快省级农产品加工园区建设，构筑“四化同步”发展新优势；大悟重点发展生态文化旅游区，建设绿色能源示范县；孝昌重点发展特色休闲农业，建设林业生态示范县。

　　（四）致力于提升生态文明，切实加强生态建设

　　切实加强环境保护。坚持绿色发展方向，把生态文明贯穿发展全过程，让孝感更加美丽、更具魅力。实施蓝天、碧水、宁静、清洁“环保四大工程”。严格执行市人大常委会《关于加强王母湖、野猪湖保护工作的决议》，实施澴东湖泊生态环境保护。推动临空经济区争创国家“绿色生态示范城区”。确保省级环保模范城创建达标。

　　切实促进资源节约。坚持低碳发展、循环发展。加快市高新区园区循环化改造，提升盐化、磷化、火电产业循环经济发展水平。充分利用再生资源，提高资源利用效率。支持企业引进先进技术，发展新兴能源产业。实施既有建筑节能改造，严格新建建筑节能监管，鼓励公共机构使用节能技术。开展国土资源节约集约模范县市创建。

　　切实强化污染治理。从源头预防、过程控制和末端治理等环节入手，全程加强污染治理。实施机动车环保标志管理，加强PM2.5监测，改善空气质量。整治主要河流沿线排污口，启动城区老澴河南岸截污管网建设，争取槐荫河、澴河水质不断改善。提升城区垃圾处理能力，启动建设垃圾焚烧发电项目。实施“千村示范万村整治”工程，控制农村面源污染。

　　（五）致力于改善民生福祉，切实加强社会建设

　　强化社会保障。加大投入力度，确保民生支出占地方公共财政预算支出的75％以上。健全城乡均等的就业培训、就业服务、就业援助三大体系，促进弱势群体充分就业。扩大五项保险覆盖面，实现社会保障从制度全覆盖向人群全覆盖转变。完善市直“三区”“三个一体化、一个新机制”，逐步向县市区开发区推广。建立城乡低保、五保供养标准自然增长机制，全面实现动态管理。完善造血式“双助”长效机制，切实保障弱势群体基本生活。推进“三房并轨”，完善住房保障制度体系。巩固完善住房公积金管理体制。

　　发展社会事业。突出教育公平，推动优质资源向农村学校和困难群体倾斜。实施第二期学前教育三年行动计划，促进义务教育均衡发展，推进普通高中教育多样化发展，支持高等院校内涵式发展，加快职教资源整合，发展成人教育,改善特殊教育办学条件。提高基本公共卫生服务均等化水平，加快城乡基层医疗卫生服务体系建设。扩大县级公立医院改革试点。完善重特大疾病保障和救助机制。完善东城区医疗卫生功能布局。稳定低生育水平，提高出生人口素质。加快孝文化与城市建设、产业发展深度融合，加强非物质文化遗产保护，继续办好“中华孝文化旅游节”，推动“中华孝文化名城”建设取得新进展。加快孝感日报传媒集团产业园区建设，组建孝感广电传媒集团。加快建设市文化中心，统筹推进市县乡文化场馆建设。促进妇女儿童事业发展，重视关爱老年人、残疾人，依法保护其合法权益。贯彻落实民族政策，依法管理宗教事务。做好第三次全国经济普查。争创“全国双拥模范城”七连冠、“全省双拥模范城”八连冠。

　　创新社会治理。坚持政府主导与社会化、市场化相结合，创新社会治理模式。加大“三类”新社区建设力度，完善3个“1+X”综合服务管理体系，推进网格化管理和小区物业管理向乡镇延伸。完成第九届村委会换届选举。深化“平安孝感”、“法治孝感”建设，深入开展“六五”普法，完善社区矫正工作机制。落实综治信访维稳“十大机制”，畅通、拓宽信访渠道。健全完善应急联动机制，提高预防和处置突发公共事件能力。落实安全生产主体责任，加强隐患排查治理，从源头预防安全事故。加强价格调控监管，保持物价总水平基本稳定。加强农村饮水安全管理。对食品药品实行最严格全过程监管，用重力、重责、重典治理，确保“舌尖上的安全”。

　　各位代表，市政府将继续办好改善民生“十件实事”。⑴新改建100个示范性城市社区居家养老服务中心（站）和农村老年人互助照料活动中心；组织孝感城区65岁以上老人免费体检。⑵启动东城区小学建设；新改建农村中小学教师保障住房500户，配套完善食宿、淋浴设施500套。⑶改扩建乡镇卫生院25个；医疗救助贫困群众2.4万人以上。⑷改建市体育中心；向社区、行政村新增体育设施320套。⑸建设100家放心粮油连锁店。⑹贯通孝感城区天仙北路、黄陂东路、航空南路等断头路；新改建垃圾中转站12座、公厕14座。⑺新建孝感城区社会公共综合停车场1处；新增公交线路2-3条，新增公交车30辆。⑻新建孝感城区2个标准化集贸市场，改造升级13个集贸市场。⑼实行残疾人市、县城区免费乘坐公交车。⑽免费办理困难群众法律援助案件1200件以上；援助3000名困难人员、高校毕业生就业创业。

　　三、切实加强政府自身建设

　　围绕建设责任型、服务型、法治型、廉洁型政府，切实加强自身建设，不断提升政府公信力和执行力。

　　1、以全市政府系统开展党的群众路线教育实践活动为契机，突出为民、务实、清廉主题，倡导“责任在我、执行在我、落实在我”理念，践行“一线工作法”，推动情况在一线摸清、问题在一线解决、工作在一线落实、发展在一线体现，打造一支政治强、责任清、作风正、工作实的公务员队伍。

　　2、强化层级担当，建立人人担当、层层担当的责任体系，打造畅通“最后一公里”落实链条，做到小事不拖拉，难事不回避，好事不揽功，大事不独断。

　　3、切实转变政府职能，抓好政府职能转变“接、放、管”，做好机构改革“控、调、改”。深化行政审批制度改革，继续取消、下放一批行政审批事项。

　　4、推行精细化管理，加强公务员能力席位建设，强化平时考核，提升行政效能，创建示范型机关。

　　5、加强市电子政务云中心建设，推动统一电子监察综合管理平台、公共资源交易平台、市县乡村四级政务服务体系规范化建设。

　　6、坚持在人大、政协监督支持下开展工作，认真执行人大及其常委会的决议决定和审议意见，重大决策出台前向人大报告、与政协协商并听取意见，提高人大代表建议和政协提案的办理质量。制定实施《孝感市加强法治政府建设规划》。

　　7、研究制定厉行勤俭节约、公务接待、公务用车等实施细则。推进预算决算信息公开和“三公”经费公开，“三公”经费支出只减不增。

　　8、加强行政执法和效能监察，加大审计监督力度,严肃查处违法违纪违规行为，严格规范公务员廉洁从政行为。

　　各位代表！建市20年来，勤劳朴实、智慧勇敢的孝感人民创造了辉煌成就，也一定能够在第三次创业伟大进程中再立新功！让我们在省委、省政府和市委的坚强领导下，凝神聚力，砥砺前行，全面推进“五个跨越”，加快建设“五个城市”，为共同谱写中国梦的“孝感篇”而努力奋斗！
